# The association of patient safety culture with intent to leave among Jordanian nurses: a cross-sectional study

**DOI:** 10.1186/s12912-023-01386-7

**Published:** 2023-06-30

**Authors:** Islam Oweidat, Ghada Abu Shosha, Kawther Dmaidi, Abdulqadir J. Nashwan

**Affiliations:** 1 Faculty of Nursing, Zarqa University, Zarqa, Jordan; 2 Nursing Department, Hamad Medical Corporation, P.O. Box 3050, Doha, Qatar

**Keywords:** Patient safety culture, Nurses, Jordan, Intent to leave

## Abstract

**Background:**

The existence of patient safety culture is crucial for healthcare providers’ retention, particularly for nurses. Patient safety culture is getting more attention from healthcare organizations worldwide, and Jordan is no exception. Nurses’ satisfaction and retention are paramount to providing safe, high-quality patient care.

**Purpose:**

To investigate the relationship between patient safety culture and intent to leave among Jordanian nurses.

**Methods:**

A descriptive cross-sectional design was used. A sample of 220 nurses was selected through convenience sampling from one governmental and one private hospital in Amman. The patient safety culture survey and anticipated turnover scale were used to collect data. Descriptive statistics and Pearson r correlation were used to answer the research questions.

**Results:**

The findings showed that nurses had 49.2% positive scores for patient safety. Teamwork (65.3%) and handoff and exchange of information (62% each) had the highest scores, while staffing and workplace (38.1%) and response to error (26.6%) had the lowest. Moreover, nurses had strong intentions to leave their jobs (M = 3.98). A moderately significant but not highly negative relationship existed between patient safety culture and intent to leave (r = -0.32, p = 0.015).

**Conclusions:**

There are opportunities to improve patient safety culture, satisfaction, and nurse retention in Jordanian hospitals by implementing several recommendations, such as ensuring better staffing patterns and increasing staff motivation by utilizing various available methods.

**Supplementary Information:**

The online version contains supplementary material available at 10.1186/s12912-023-01386-7.

## Introduction

Nurses play a crucial role in healthcare organizations as they make up the majority of healthcare professionals and are responsible for providing and coordinating healthcare activities among other professionals [1]. They are vital in ensuring patient safety in hospitals and medical centers, contributing to the improvement of care quality through the provision of effective and safe care, monitoring quality and patient safety indicators, incident reporting, and risk assessment and management [2]. However, in high-stress work environments characterized by increasing patient volume and acuity, nurses often report unsafe working conditions, burnout, job dissatisfaction, and a desire to leave their jobs [3]. Previous studies have shown a connection between patient safety culture and intent to leave, but further exploration of this association is needed within the Jordanian healthcare sector, particularly among Jordanian nurses.

Patient safety has gained significant attention as a global concern in the healthcare industry. Patient safety culture refers to the shared values, attitudes, and behaviors within an organization that prioritize patient safety and the minimization of harm [4]. It is focused on preventing unintentional harm or injury to patients during the delivery of healthcare services [1]. Unsafe medical practices have been identified as a major source of morbidity and mortality worldwide, although the scale of the problem varies. Many people are susceptible to debilitating injuries or death directly related to medical care [2]. Consequently, patient safety has become a cornerstone of healthcare quality, prompting numerous projects and initiatives. A broader concept directly related to safe patient care is the “patient safety culture.“

Intent to leave work is influenced by various dynamic factors, including organizational factors (status, climate, culture, support, and leadership practices), work-related factors (autonomy, salaries, empowerment, and the ability to make an impact), and demographic factors (years of experience, age, and work area) [3]. Nurse turnover can result in organizational loss of knowledge and experience, decreased morale, increased workloads, and early departures from the profession [5]. This compromises continuity of care, leading to longer lengths of stay and higher recruitment and training cost [5].

Organizational safety culture has a significant impact on the provision of high-quality healthcare services [5]. Satisfied employees are more likely to retain their jobs and enhance their performance [6]. Ultimately, this contributes to a stronger healthcare system and enhances the reputation of the healthcare institution. Nurses, in particular, are central to healthcare services as they directly care for patients and act as liaisons between different hospital departments. Therefore, numerous studies have been conducted over the past decade to assess patient safety culture both regionally and internationally [5, 7, 8].

It is evident that nurse turnover is one of the major challenges faced by healthcare institutions. The primary objective of any healthcare organization is to provide optimal quality care while ensuring patient safety. This can be best achieved with well-trained and experienced nurses. Consequently, healthcare organizations must strive to maintain low turnover rates to ensure continuity in delivering high-quality care. The literature highlights several factors associated with nurses’ intent to leave, including job satisfaction, leadership support, work environment, and peer support [9].

### Significance of the study

Nurses play a vital role in the success of healthcare systems, making it essential to understand their needs, perceptions, attitudes, stressors, and their interactions within their work environment [10]. This study provides valuable knowledge and insights for hospital and nursing management, enabling them to identify important factors that contribute to nurses’ satisfaction and their willingness to continue their careers within the organization. It also offers frontline nurses a better understanding of the factors that influence their satisfaction and retention within the hospital.

Furthermore, this study represents the first investigation into the relationship between safety culture and nurses’ intent to leave work among Jordanian nurses. This valuable research has implications for nursing educators who can utilize the study’s findings to develop curricula that incorporate patient safety principles and organizational cultures that attract and retain nurses. The study’s significant contribution lies in its ability to establish the relationship between patient safety culture and nurses’ intent to leave, assisting nurse managers in making informed decisions to effectively manage high turnover rates and ensure the delivery of high-quality care for patients and their families.

Ultimately, the results of this study benefit healthcare recipients by maintaining a safe environment for their care and ensuring the presence of qualified nurses who have a genuine intention to stay and provide them with high-quality care.

### Purpose and aim

The current study aimed to explore the association between patient safety culture and intent to leave among Jordanian nurses working in governmental and private hospitals.

### Research questions

1- What are the nurses’ perceptions regarding the level of patient safety culture and intent to leave?

2- Is there a relationship between patient safety culture and intent to leave among Jordanian nurses?

### Conceptual Framework

The current study was guided by a conceptual framework that was developed by the researchers and based on the previous literature; in which the independent variable was patient safety culture and the independent variable was intent to leave, whereas the moderators were extracted from the literature as shown in the diagram below.

**Figure:**
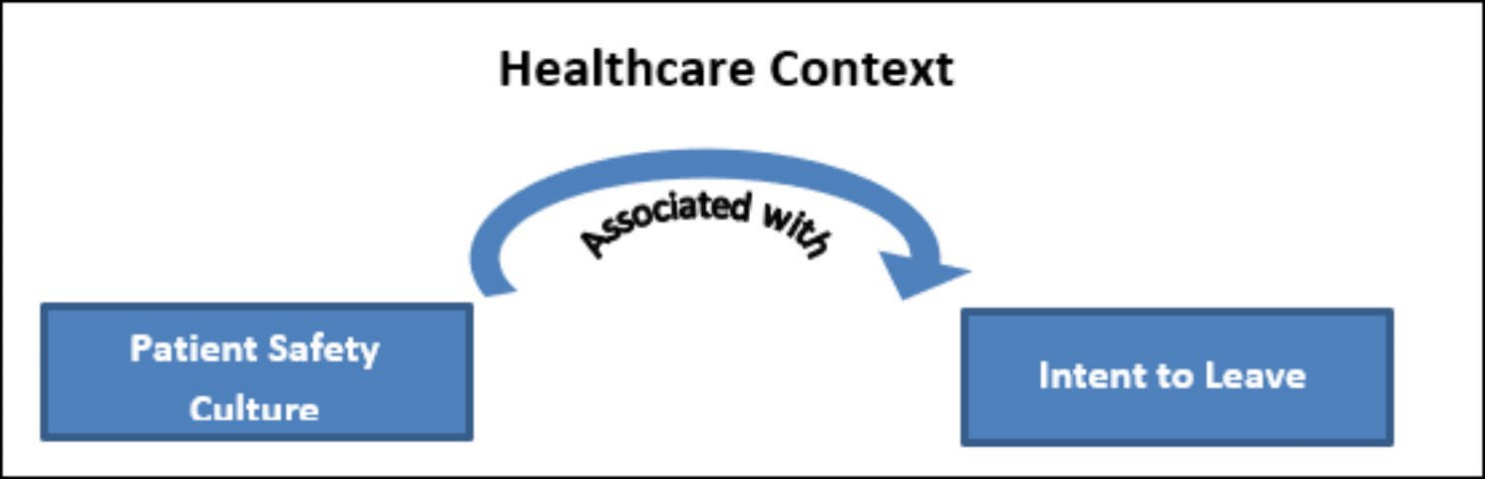
Conceptual framework of the study

Extensive research has been conducted on the perceptions of patient safety culture and intent to leave among nurses in the healthcare field. However, few studies, especially in Jordan, have specifically investigated the association between patient safety culture and intent to leave among nurses who directly provide care to patients and their families. In a previous Iranian study, the hospital survey on patient safety culture (HSOPSC) was used to assess the association between patient safety culture and job stress among nurses. The study revealed average to high levels of stress among nurses, with organizational learning scoring the highest and handoffs and transitions scoring the lowest among patient safety culture dimensions. The findings also indicated a significant relationship between safety culture and nurses’ stress within healthcare organizations, suggesting the need for upper management to prioritize safety culture and monitor and evaluate it to enhance patient safety and reduce hospital stress [1].

Another recent study conducted by Al-Surimi et al. (2022) aimed to explore the impact of patient safety culture on job satisfaction and intention to leave among healthcare workers in the Middle East. The study found that two-thirds of the participants reported a very good patient safety grade, and approximately three-quarters of them had no intention to leave their jobs. The domains of staffing and hospital management support for patient safety were associated with higher odds ratios for job satisfaction, while the composites of teamwork within the hospital unit and hospital management support for patient safety were significantly associated with lower intent to leave [11].

A systematic review study conducted in Portugal aimed to review findings related to the safety culture dimensions identified in the HSOPSC and their potential contribution to improving the quality and safety of hospital services. The results revealed that teamwork within units and organizational learning-continuous improvement were the strongest reported dimensions, while non-punitive response to error, staffing, handoffs and transitions, and teamwork across units were identified as the weakest dimensions. Overall, the study suggested that safety culture assessments should be linked with improvement strategies and managerial support to achieve better outcomes, as patient safety cultures in the participating hospitals were often underdeveloped or weak [8].

The literature indicates that intent to leave among nurses is influenced by various factors, such as working hours. Jarrar et al. (2023) found a significant association between working hours duration and nurses’ intention to leave their jobs [12].

Lastly, a Jordanian study aimed to investigate the relationship between nurses’ work environment, job satisfaction, and their intent to stay in hospital settings. The results demonstrated a significant positive relationship between the nursing work environment and job satisfaction, as well as between the work environment and intent to stay. Furthermore, the work environment was found to significantly predict nurses’ job satisfaction and intent to stay in the hospital.

## Methods

The study employed a descriptive cross-sectional correlational design and was conducted in two hospitals, one governmental and one private, located in Amman, the capital of Jordan. These hospitals were chosen due to their comprehensive nature, referral status, and provision of different levels of care. To ensure comprehensive reporting of the cross-sectional study, the researchers followed the STROBE guidelines.

Convenience sampling with specific inclusion and exclusion criteria was utilized to select eligible nurses who were available at the study sites to participate in the survey questionnaires. Convenience sampling was chosen to facilitate easy access to potential participants compared to random selection, and it allowed the researchers to invite eligible and available participants considering the time constraints and heavy patient workload. The sample size was determined using a sample size calculator, taking into account a 95% confidence level and a 5% margin of error. With an estimated population size of around 500 nurses, a sample size of 220 nurses was deemed appropriate for achieving generalizability of the study results.

The inclusion criteria were as follows: (1) holding at least a bachelor’s degree, (2) having a minimum of one year of experience in the current work settings to ensure familiarity with the organizational culture, and (3) being a registered nurse in Jordan. The exclusion criteria involved occupying a managerial position or having a role in the managerial track, such as quality and infection control departments.

### Study Instruments

Data collection instruments that were used in this study are (1) a Socio-demographic sheet; (2) (HSOPSC) version 2; (3) an Anticipated Turnover Scale (ATS).

Socio-demographic Sheet includes age, gender, marital status, educational level, hospital type, work unit, shift rotation, length of experience in the hospital, length of experience in the unit, and the number of working hours per week.

### HSOPSC (version 2.0)

HSOPSC version 2.0 is an update of the original Hospital Survey (1.0) developed by the Agency for Healthcare Research and Quality (AHRQ) in 2004. The HSOPSC is a validated scale that measures the efficiency of the work environment regarding preventing errors that lead to undesirable events [13]. It focuses on patient safety and error and event reporting. There are 10 composite measures by 32 items. Additionally, the survey includes one question that asks respondents to indicate the number of events they reported over the past 12 months. The majority of responses can be answered on 6 points Likert scale. Finally, the survey contains six items asking about respondent characteristics such as; staff position, unit/work area, hospital tenure, unit/work area tenure, work hours, and interaction with patients).

The percentage of the positive score is calculated based on the sum of the strongly agree and agree on answers for positively stated items divided by the total number of answers for that item, and for strongly disagree and disagree answers for negatively stated items divided by the total number of answers for that item. The average score of items under a certain composite is calculated to represent the overall score of that composite [8].

### ATS

ATS was developed by Hinshaw & Atwood in 1984. The ATS consists of 12 items that measure intent to leave on a 7-point Likert scale. The seven options in the ATS are agreed strongly (AS), moderately agree (MA), slightly agree (SA), uncertain (U), slightly disagree (SD), moderately disagree (MD), and disagree. In addition, items are either positively or negatively stated. Accordingly, items (2, 4, 5, 7, 11, and 12) are positively stated, while (1, 3, 6, 8, 9, and 10) are negatively stated. Two more questions concern the intention to leave their position in the next 6 months and the intention to leave the nursing profession in the next 6 months. A meta-analysis of studies of registered nurses maintained the ATS’s validity and reliability. A mean weighted effect size of reliability across 12 studies was Cronbach’s alpha = 0.89, while construct validity correlating ATS and four job satisfaction measures in 7 studies was found to be 0.53 [14].

### Ethical considerations

The official approvals to conduct the study were obtained from the institutional review board (IRB) of the Faculty of Nursing at Zarqa University and the selected settings’ administrative authorities. Additionally, a consent form and written explanation were distributed online for participants that clarified the study objectives and procedure for them to avoid ethical concerns regarding enrolling participants in the study against his/her willingness. Moreover, the participants were assured that all the collected data will be kept confidential and anonymous on the researcher’s own computer which has a password. The researcher also informed the participants that no risk was to be handled during this study. Finally, the permission to use the tools were obtained from the primary authors.

### Data Collection Procedure

To ensure efficient data collection, the researchers followed a specific process. First, they obtained approval from the head nurses of the selected departments to conduct the study. With their permission, the researchers requested a list of phone numbers for the nurses working in those departments. In order to maintain data confidentiality, each participant was assigned a unique ID number, which was associated with their phone number. The researchers obtained explicit consent from the participants before proceeding.

To distribute the survey, the researchers utilized the WhatsApp application. They sent the link to an online questionnaire package, designed using Google Forms, which included the consent form and the study instruments. Before the actual data collection, the researchers ensured the survey’s readability and clarity by piloting it on a 10% sample size, which was not included in the final study to avoid bias in responses.

Participants completed the questionnaires online, and their responses were directly recorded on the designated online form, ensuring real-time data storage. Completed questionnaires were subsequently reviewed to ensure they met the study’s inclusion criteria and were complete. The collected data were then analyzed using the Statistical Package for the Social Sciences (SPSS) for further analysis.

The data collection process was conducted between April 28, 2020, and June 25, 2020, within the selected hospitals.

### Data Analysis

Data were analyzed using the (SPSS), Version 26. Frequencies and percentages, means and standard deviation were performed to describe study variables including demographic variables, patient safety culture, and intent to leave among nurses. Pearson’s r coefficient correlation was used to determine the presence of a relationship between patient safety culture, and intent to leave. Additionally, all the assumptions were checked and met before running the needed statistical analysis. In order to avoid duplication or fraud in the online survey; each respondent was provided with a unique link to access the survey, so this ensures that only one response is received per unique link and eliminates the possibility of duplicated responses.

Besides, in order to analyze potential non-response bias; researcher increased responses rates by using follow-up emails with all eligible participants. Moreover, to analyze any potential early versus late biases; the researcher had monitored the response rates of the survey to identify if there were any significant differences in response pattern. All the responses that have more than 20% missing values were not accepted to be analyzed, which they count about five responses. Lastly, in order to mitigate common method bias, separating the measurement of the predictor and outcome variables were assured by the researcher.

## Results

A total of 220 nurses participated in this study, resulting in a response rate of 90%. The participants’ demographic characteristics are presented in Table 1. The average age of the participants was 31.99 years (SD = 6.88). The majority of the participants were female (n = 133, 60.5%), and more than half of them were married (n = 125, 56.8%). Around three-fourths of the sample held a bachelor’s degree (n = 166, 75.5%). In terms of years of experience in the hospital, approximately half of the participants (n = 109, 49.5%) had worked for 1 to 5 years, while 23.2% (n = 51) had worked for 6 to 10 years, 15.5% (n = 34) had 11 or more years of experience, and 11.8% (n = 26) had less than a year of experience.

**Table 1:** Demographic Characteristics of the participants (N = 220)

Category	Sub-category	Freq.	Perc.	Mean (STD)
Age				31.99 (6.88)
Gender	Male	87	39.5%	
	Female	133	60.5%	
Marital Status	Single	86	39.1%	
	Married	125	56.8%	
	Divorced	8	3.6%	
	Widowed	1	0.5%	
Educational Level	Bachelor’s degree	166	75.5%	
	Master’s degree	46	20.9%	
	Doctoral Degree	8	3.6%	
Hospital Type	Governmental	114	51.8	
	Private	106	48.2	
Work unit	ER	48	21.8%	
	ICU-CCU	45	20.5%	
	Medical-surgical ward	80	36.4%	
	OR	12	5.5%	
	Endoscopy	9	4.1%	
	Post-natal, NICU	26	11.8%	
Shift Rotation	Yes	181	82.3%	
	No	39	17.7%	
How long have you worked in a hospital	Less than 1 year	26	11.8%	
	1 to 5 years	109	49.5%	
	6 to 10 years	51	23.2%	
	11 or more years	34	15.5%	
How long have you worked in the unit	Less than 1 year	31	14.1%	
	1 to 5 years	99	45%	
	6 to 10 years	58	26.4%	
	11 or more years	32	14.5%	
Typically, how many hours per week do you work in this hospital?	Less than 30 h per week	10	4.5%	
	30 to 40 h per week	99	45%	
	More than 40 h per week	111	50.5%	

Table 2 displays the percentages of positive scores for different items and composites based on the analysis guidelines provided by AHRQ. The overall average of positive scores for all patient safety culture composites was 49.2%. The highest score was observed for the teamwork composite (65.3%), followed by handoff and exchange of information (62.0%). In contrast, the lowest scores were recorded for staffing and work pace (38.1%) and response to error (26.6%). Regarding the number of events reported in the past 12 months, 37.2% (n = 82) reported 1 to 2 events, 32.7% (n = 72) reported no events, 20.9% (n = 46) reported 3 to 5 events, 5% (n = 11) reported 11 or more events, and 4.1% (n = 9) reported 6 to 10 events. The overall mean score of patient safety culture (HSOPSC) was 3.24 (SD = 1.14), with the highest-scoring items being “In this unit, we work together as an effective team” (mean value = 3.89) and “The actions of hospital management show that patient safety is a top priority” (mean value = 3.80). The mean score of patient safety culture is illustrated in Table 3.

**Table 2:** Patient Safety Culture Composites Percent Positive Score

Composite	For Positively Worded Items, # of “Strongly agree” or “Agree” Responses	For Negatively Worded Items, # of “Strongly disagree” or “Disagree” Responses	Total # of Responses to Item (Excluding Missing and Does not apply/Don’t know Responses)	Percent of Positive Response to Item
**Teamwork**				
A1-positively worded: “In this unit, we work together as an effective team.“	174	NA	220	79.1%
Item A8-positively worded: “During busy times, staff in this unit help each other.“	152	NA	220	69.1%
Item A9-negatively worded: “There is a problem with disrespectful behavior by those working in this unit.“	NA	105	220	47.7%
***Average percent positive response across the 3 items***				**65.3%**
**Staffing and Work Pace**				
A2. In this unit, we have enough staff to handle the workload.	68	NA	218	31.2%
A3. Staff in this unit work longer hours than is best for patient care. (negatively worded)	NA	65	220	29.5%
A5. This unit relies too much on temporary, float, or PRN staff. (negatively worded)	NA	120	220	54.5%
A11. The work pace in this unit is so rushed that it negatively affects patient safety. (negatively worded)	NA	82	220	37.3%
***Average percent positive response across the 4 items***				**38.1%**
**Organizational Learning- Continuous improvement**				
A4. This unit regularly reviews work processes to determine if changes are needed to improve patient safety.	121	NA	218	55.5%
A12. In this unit, changes to improve patient safety are evaluated to see how well they work.	126	NA	217	58.1%
A14. This unit lets the same patient safety problems keep happening. (negatively worded)	NA	87	220	39.5%
***Average percent positive response across the 3 items***				**51.0%**
**Response to Error**				
A6. In this unit, staff feels like their mistakes are held against them. (negatively worded)	NA	44	219	20.1%
A7. When an event is reported in this unit, it feels like the person is being written up, not the problem. (negatively worded)	NA	45	220	20.5%
A10. This unit focuses on learning rather than blaming individuals when staff makes errors.	83	NA	216	38.4%
A13. In this unit, there is a lack of support for staff involved in patient safety errors. (negatively worded)	NA	60	220	27.3%
***Average percent positive response across the 4 items***				**26.6%**
**Supervisor, Manager, or Clinical Leader Support**				
B1. My supervisor, manager, or clinical leader seriously considers staff suggestions for improving patient safety.	94	NA	215	43.7%
B2. My supervisor, manager, or clinical leader wants us to work faster during busy times, even if it means taking shortcuts. (negatively worded)	NA	84	220	38.2%
B3. My supervisor, manager, or clinical leader takes action to address patient safety concerns that are brought to their attention.	122	NA	213	57.3%
***Average percent positive response across the 3 items***				**46.4%**
**Communication about Errors**				
C1. We are informed about errors that happen in this unit.	109	NA	218	50.0%
C2. When errors happen in this unit, we discuss ways to prevent them from happening again.	109	NA	217	50.2%
C3. In this unit, we are informed about changes based on event reports.	124	NA	216	57.4%
***Average percent positive response across the 3 items***				**52.5%**
**Communication Openness**				
C4. In this unit, the staff speaks up if they see something that may negatively affect patient care.	128	NA	216	59.3%
C5. When the staff in this unit see someone with more authority doing something unsafe for patients, they speak up.	110	NA	210	52.4%
C6. When the staff in this unit speak up, those with more authority are open to their patient safety concerns.	102	NA	212	48.1%
C7. In this unit, the staff are afraid to ask questions when something seems wrong. (negatively worded)	NA	75	216	34.7%
***Average percent positive response across the 4 items***				**48.6%**
**Reporting Patient Safety Event**				
D1. When a mistake is caught and corrected before reaching the patient, how often is this reported?	93	NA	211	44.1%
D2. When a mistake reaches the patient and could have harmed the patient but did not, how often is this reported?	126	NA	211	59.7%
***Average percent positive response across the 2 items***				**50.8%**
**Hospital Management Support for Patient Safety**				
F1. The actions of hospital management show that patient safety is a top priority.	149	NA	215	69.3%
F2. Hospital management provides adequate resources to improve patient safety.	96	NA	212	45.3%
F3. Hospital management seems interested in patient safety only after an adverse event happens. (negatively worded)	NA	79	218	36.2%
***Average percent positive response across the 3 items***				**50.3%**
**Handoffs and Information Exchange**				
F4. Important information is often left out when transferring patients from one unit to another. (negatively worded)	NA	144	220	65.5%
F5. During shift changes, important patient care information is often left out. (negatively worded)	NA	141	220	64.1%
F6. During shift changes, there is adequate time to exchange all key patient care information.	122	NA	216	56.5%
***Average percent positive response across the 3 items***				**62.0%**
***The overall average of positive scores of all composites of patient safety culture***				**49.2%.**

**Table 3:** Patient Safety Culture Survey Item Analysis

Item	Mean	STD
In this unit, we work together as an effective team	3.89	0.975
During busy times, staff in this unit help each other	3.66	1.200
There is a problem with disrespectful behavior by those working in this unit	3.18	1.187
In this unit, we have enough staff to handle the workload	2.55	1.339
Staff in this unit work longer hours than is best for patient care	2.61	1.159
This unit relies too much on temporary, float, or PRN staff	3.30	1.146
The work pace in this unit is so rushed that it negatively affects patient safety	2.88	1.180
This unit regularly reviews work processes to determine if changes are needed to improve patient safety	3.29	1.196
In this unit, changes to improve patient safety are evaluated to see how well they worked	3.43	1.135
This unit lets the same patient safety problems keep happening	2.96	1.106
In this unit, staff feel like their mistakes are held against them	2.40	1.118
When an event is reported in this unit, it feels like the person is being written up, not the problem	2.40	1.100
When staff make errors, this unit focuses on learning rather than blaming individuals	2.91	1.337
In this unit, there is a lack of support for staff involved in patient safety errors	2.63	1.137
My supervisor, manager, or clinical leader seriously considers staff suggestions for improving patient safety	3.07	1.317
My supervisor, manager, or clinical leader wants us to work faster during busy times, even if it means taking shortcuts	2.85	1.165
My supervisor, manager, or clinical leader takes action to address patient safety concerns that are brought to their attention	3.47	1.128
We are informed about errors that happen in this unit	3.60	1.096
When errors happen in this unit, we discuss ways to prevent them from happening again	3.40	1.280
In this unit, we are informed about changes that are made based on event reports	3.67	1.191
In this unit, staff speak up if they see something that may negatively affect patient care	3.72	1.116
When staff in this unit see someone with more authority doing something unsafe for patients, they speak up	3.60	1.218
When staff in this unit speak up, those with more authority are open to their patient safety concerns	3.53	1.160
In this unit, staff are afraid to ask questions when something does not seem right	3.13	1.212
When a mistake is caught and corrected before reaching the patient, how often is this reported?	3.40	1.255
When a mistake reaches the patient and could have harmed the patient but did not, how often is this reported?	3.79	1.039
The actions of hospital management show that patient safety is a top priority	3.80	1.011
Hospital management provides adequate resources to improve patient safety	3.26	1.202
Hospital management seems interested in patient safety only after an adverse event happens	3.02	1.018
When transferring patients from one unit to another, important information is often left out	3.50	0.948
During shift changes, important patient care information is often left out	3.49	0.934
During shift changes, there is adequate time to exchange all key patient care information	3.38	1.089
**The overall mean score of patient safety culture**	**3.24**	**1.14**

Table 4 presents the item-level analysis for the anticipated turnover scale. The overall average for the scale indicated that nurses, on average, had an intention to leave their jobs (M = 3.98, SD = 1.70). The highest-scoring items were item #9, “I don’t have any specific idea how much longer I will stay” (M = 5.08, SD = 1.43), and item #8, “I am certain I will be staying here a while” (M = 5.00, SD = 1.39). On the other hand, the lowest-scoring items were item #11, “There are big doubts in my mind as to whether or not I will stay in this agency” (M = 3.19, SD = 1.68), and item #2, “I am quite sure I will leave my position in the foreseeable future” (M = 2.96, SD = 1.71).

**Table 4:** Anticipated Turnover Scale item analysis

#	Item	Mean	STD
1	I plan to stay in my position awhile. (R)	4.78	1.74
2	I am quite sure I will leave my position in the foreseeable future.	2.96	1.71
3	Deciding to say or leave my position is not a critical issue for me at this point in time.	4.77	1.73
4	I know whether or not I’ll be leaving this agency within a short time.	3.61	1.73
5	If I got another job offer tomorrow, I would consider it seriously.	2.61	1.73
6	I have no intentions of leaving my present position. (R)	4.27	1.96
7	I’ve been in my position about as long as I want to. (R)	3.43	1.78
8	I am certain I will be staying here awhile (R)	5.00	1.39
9	I don’t have any specific idea how much longer I will stay.	5.08	1.43
10	I plan to hang on to this job for a while. (R)	4.65	1.72
11	There are big doubts in my mind as to whether or not I will stay in this agency.	3.19	1.68
12	I plan to leave this position shortly.	3.42	1.82
	**Overall**	**3.98**	**1.70**

Regarding the intent to leave within six months, the majority of participants (n = 136, 61.8%) indicated that they did not intend to leave their positions, while 21.4% (n = 47) expressed an intention to leave. For the intention to leave the nursing profession within six months, the majority of participants (n = 173, 78.6%) intended to stay, while 21.4% (n = 47) expressed an intention to leave. Table 5 illustrates the intention to leave within six months.

**Table 5:** Intention to leave within six months

Item		Freq.	Perc.
Do you intend to leave your position in the next 6 months?	Yes	84	38.2
	No	136	61.8
Do you intend to leave the nursing profession in the next 6 months?	Yes	47	21.4
	No	173	78.6

A Pearson product-moment correlation revealed a moderate negative relationship between patient safety culture and anticipated turnover (r = -0.32, p = 0.015), as shown in Table 6.

**Table 6:** Relationship between patient safety culture and anticipated turnover

Scales	Mean	STD	r	p
Patient safety culture	3.24	1.14	-0.32	0.015
Anticipated turnover scale	3.98	1.70		

In conclusion, this study revealed variations in nurses’ perceptions of patient safety culture composites, with teamwork being perceived as the most positive, followed by handoff and exchange of information. Conversely, supervisor, manager, or clinical leader support and response to error scored lower, indicating a culture of blame and a lack of managerial support for a safety culture. The overall average for anticipated turnover indicated a strong willingness to leave their jobs among nurses. However, the majority of participants reported no intention to leave their workplace within the next six months. The tests of relationships showed a significant association between the overall average of safety culture composites and intention to leave work.

## Discussion

The objective of this study was to analyze the perceptions of patient safety culture and its association with intent to leave among Jordanian nurses. The findings of this study contribute to the existing literature by demonstrating the relationship between patient safety culture and intent to leave. The results suggest that improving patient safety culture is essential in promoting nurses’ intention to stay and reducing turnover. Establishing leadership that supports patient safety culture is crucial in fostering a magnet environment where nurses are more likely to remain in their positions.

In terms of patient safety culture composites, the results of this study revealed that teamwork and handoff and exchange of information received the highest scores. Conversely, staffing and workplace and response to error received the lowest scores. These findings are consistent with a previous Jordanian study by Ammouri et al. (2015), which used the HSOPSC (Version one) and reported similar results. In both studies, teamwork was perceived as the most positive composite, while response to error and staffing scored the lowest [5]. However, it should be noted that differences in the tool used may contribute to variations in the findings.

In contrast, a study conducted in Iran (2017) with a sample of 380 nurses found that organizational learning received the highest score, while handoffs and transitions of care received the lowest score. The overall mean score for safety culture composites was similar to our study. However, differences in the settings of the studies may account for variations in composite-level results [1].

Furthermore, a randomized controlled trial study conducted in Iran found that organizational learning and teamwork scored the highest, while non-punitive response to error and staffing scored the lowest. The study demonstrated a significant improvement in the overall score of safety culture and some dimensions after implementing a nursing empowerment program. These findings align with our results, highlighting the importance of staff support and a blame-free culture in response to errors to enhance safety culture [15].

Regarding intent to leave, our study revealed that nurses expressed a strong intention to leave their jobs. This aligns with the findings of Lagerlund et al. (2015), which explored the relationship between perceived leadership and intent to leave among nurses. One-third of the nurses in their study intended to leave their work within 12 months. Factors such as fewer years of experience, inadequate work-related education, higher burnout, and lower scores of leadership perceptions were associated with intent to leave [2, 3]. In our study, the majority of participants intended to leave their jobs within the next year, although the specific reasons behind these intentions were not explored.

Furthermore, a study conducted in Jordan found that a significant proportion of nurses intended to leave their jobs due to factors such as pay and benefits, nurse-to-bed ratio, and lack of recognition. Dissatisfaction stemming from low pay, work overload, and lack of recognition contributed to their intent to leave [16]. These findings support our results, indicating a strong intent among Jordanian nurses to leave their jobs and nursing careers due to work pressures, low pay, and lack of support and recognition.

Additionally, a study in Thailand revealed that nurses working in environments with better conditions had lower burnout scores, dissatisfaction, and intention to leave. Factors such as nursing foundation of quality of care, nurse managerial ability, leadership and support, nurse perception of hospital affairs, collegial nurse-physician relations, and staff resource and adequacy were identified as key environmental conditions that influenced nurses’ retentions [17].

The findings of our study indicate a moderate negative relationship between patient safety culture and anticipated turnover. These results are in line with previous studies that have demonstrated a negative correlation between turnover intention and safety climate [18]. Similar associations between intent to stay, safety culture features, and organizational safe behaviors have also been reported [19, 20].

In conclusion, this study highlights the importance of patient safety culture in relation to nurses’ intent to leave their jobs. The findings suggest that efforts to improve patient safety culture and address the factors contributing to turnover intention are crucial. Hospital management should collaborate with department managers and unit heads to identify and address the reasons behind nurses’ intention to leave, aiming to improve retention. Additionally, establishing magnet hospitals, strengthening organizational culture, and addressing nurses’ concerns are vital steps toward reducing the desire of nurses to leave their jobs.

### Implications

The results of the current study have important implications for nursing practice; in which nurses should understand that patient safety culture is vital to improve satisfaction and willingness to stay at work. Thus, they can help to improve safety culture by reporting safety-related incidents, speaking up, and communicating about any issues at work. Additionally, nursing management should work closely with hospital management to improve the safety culture, as well as hospital and nursing management should do regular rounds that aim to speak to staff, discuss work-related issues, and show interest in the health and wellbeing of the staff as well, which reflects their support and involvement.

Moreover, methods for motivating the staff to perform at or above the expected level of performance should be provided, such as: increasing remuneration, providing non-monetary incentives such as acknowledgement and recognition, and observing nurses’ needs. Lastly, future studies should take qualitative approach to explore managers’ experiences with actions promoting safety culture, and retention, to have a more comprehensive understanding of these concepts and their relationships.

### Strength and Limitations

Despite the study strength in the appropriateness of study design to purpose, homogeneity of study settings and its novelty, the findings of this study are subject to several limitations. A methodological limitation might be that surveys only provide a snapshot of the safety culture, and intent to leave through measuring perceptions of nurses during one period. They may provide a limited understanding about aspects of organizational safety culture. Adding to that, the study design was descriptive; there was no intervention employed that will aim at improving the safety culture of the nurses, and cross-sectional; it did not attempt to measure safety culture after follow-ups or over a period. Generalization of the study results is limited to nurses from the participating hospitals considering the factors that make one hospital institution different from another. Moreover, sample size was relatively small; larger sample size could increase the strength and generalizability of the findings.

## Conclusion

In light of the above findings, there is a significant negative relationship between patient safety culture and intention to leave. Based on the participants’ answers, there are opportunities to improve the safety culture and nurses’ retention in Jordanian hospitals by considering the implementation of several recommendations, including but not limited to providing continuing education programs, improving managerial commitment and support, developing administrative policies to improve staff outcomes and increasing staff motivation through the utilization of various available methods; such as recognition of early catchers for near misses, safety heroes, paying more salaries and incentives to nurses, simple thank you messages for them and establishing a supportive organizational cultures that magnet them at their organizations.

## Electronic supplementary material

Below is the link to the electronic supplementary material.

Supplementary Material 1

## Data Availability

All data generated or analyzed during this study are included in this published article.
