# Supplementary material for: The association of patient safety culture with intent to leave among Jordanian nurses: a cross-sectional study
Source: BMC Nurs. 2023 Jun 30;22:227. doi: 10.1186/s12912-023-01386-7 (PMC10311745; doi:10.1186/s12912-023-01386-7)
Supplement: Supplementary file 1 — Supplementary Material 1 [file 12912_2023_1386_MOESM1_ESM.docx]

**Appendices**

**Appendix (A): Patient Safety Culture Composite Measures**

**Appendix (B): Hospital Survey on Patient Safety Culture HSOPSC version 2.0**

**How much do you agree or disagree with the following statements about your unit/work area?**

| Does Not Apply or Don’t Know | Strongly Agree | Agree | Neither Agree nor Disagree | Disagree | Strongly Disagree | Statement |
| --- | --- | --- | --- | --- | --- | --- |
| 6 | 5 | 4 | 3 | 2 | 1 | 1.In this unit, we work together as an effective team |
| 6 | 5 | 4 | 3 | 2 | 1 | 2.In this unit, we have enough staff to handle the workload |
| 6 | 5 | 4 | 3 | 2 | 1 | 3.Staff in this unit work longer hours than is best for patient care |
| 6 | 5 | 4 | 3 | 2 | 1 | 4.This unit regularly reviews work processes to determine if changes are needed to improve patient safety |
| 6 | 5 | 4 | 3 | 2 | 1 | 5.This unit relies too much on temporary, float, or PRN staff |
| 6 | 5 | 4 | 3 | 2 | 1 | 6.In this unit, staff feel like their mistakes are held against them |
| 6 | 5 | 4 | 3 | 2 | 1 | 7.When an event is reported in this unit, it feels like the person is being written up, not the problem |
| 6 | 5 | 4 | 3 | 2 | 1 | 8.During busy times, staff in this unit help each other |
| 6 | 5 | 4 | 3 | 2 | 1 | 9.There is a problem with disrespectful behavior by those working in this unit |
| 6 | 5 | 4 | 3 | 2 | 1 | 10.When staff make errors, this unit focuses on learning rather than blaming individuals |
| 6 | 5 | 4 | 3 | 2 | 1 | 11.The work pace in this unit is so rushed that it negatively affects patient safety |
| 6 | 5 | 4 | 3 | 2 | 1 | 12.In this unit, changes to improve patient safety are evaluated to see how well they worked |
| 6 | 5 | 4 | 3 | 2 | 1 | 13.In this unit, there is a lack of support for staff involved in patient safety errors |
| 6 | 5 | 4 | 3 | 2 | 1 | 14.This unit lets the same patient safety problems keep happening |
| 6 | 5 | 4 | 3 | 2 | 1 | 15.My supervisor, manager, or clinical leader seriously considers staff suggestions for improving patient safety |
| 6 | 5 | 4 | 3 | 2 | 1 | 16.My supervisor, manager, or clinical leader wants us to work faster during busy times ,even if it means taking shortcut |
| 6 | 5 | 4 | 3 | 2 | 1 | 17.My supervisor, manager, or clinical leader takes action to address patient safety concerns that are brought to their attention |
| 6 | 5 | 4 | 3 | 2 | 1 | 18.The actions of hospital management show that patient safety is a top priority |
| 6 | 5 | 4 | 3 | 2 | 1 | 19.Hospital management provides adequate resources to improve patient safety |
| 6 | 5 | 4 | 3 | 2 | 1 | 20.Hospital management seems interested in patient safety only after an adverse event happens |
| 6 | 5 | 4 | 3 | 2 | 1 | 21.When transferring patients from one unit to another, important information is often left out |
| 6 | 5 | 4 | 3 | 2 | 1 | 22. During shift changes, important patient care information is often left out |
| 6 | 5 | 4 | 3 | 2 | 1 | 23.During shift changes, there is adequate time to exchange all key patient care information |

**How often do the following things happen in your unit/work area?**

**Think about your unit/work area:**

| Does Not Apply or Don’t Know | Always | Most of the Time | Some-times | Rarely | Never | Statement |
| --- | --- | --- | --- | --- | --- | --- |
| 6 | 5 | 4 | 3 | 2 | 1 | 1.We are informed about errors that happen in this unit |
| 6 | 5 | 4 | 3 | 2 | 1 | 2.When errors happen in this unit, we discuss ways to prevent them from happening again |
| 6 | 5 | 4 | 3 | 2 | 1 | 3. In this unit, we are informed about changes that are made based on event reports. |
| 6 | 5 | 4 | 3 | 2 | 1 | 4. In this unit, staff speak up if they see something that may negatively affect patient care. |
| 6 | 5 | 4 | 3 | 2 | 1 | 5. When staff in this unit see someone with more authority doing something unsafe for patients, they speak up. |
| 6 | 5 | 4 | 3 | 2 | 1 | 6. When staff in this unit speak up, those with more authority are open to their patient safety concerns. |
| 6 | 5 | 4 | 3 | 2 | 1 | 7. In this unit, staff are afraid to ask questions when something does not seem right. |
| 6 | 5 | 4 | 3 | 2 | 1 | 8. When a mistake is caught and corrected before reaching the patient, how often is this reported? |
| 6 | 5 | 4 | 3 | 2 | 1 | 9. When a mistake reaches the patient and could have harmed the patient, but did not, how often is this reported? |

**1. In the past 12 months, how many patient safety events have you reported?**

1. a. None
2. b. 1 to 2
3. c. 3 to 5
4. d. 6 to 10
5. e. 11 or more

**2. How long have you worked in this hospital?**

1. a. Less than 1 year
2. b. 1 to 5 years
3. c. 6 to 10 years
4. d. 11 or more years

**3. In this hospital, how long have you worked in your current unit/work area?**

1. a. Less than 1 year
2. b. 1 to 5 years
3. c. 6 to 10 years
4. d. 11 or more years

**4. Typically, how many hours per week do you work in this hospital?**

1. a. Less than 30 hours per week
2. b. 30 to 40 hours per week
3. c. More than 40 hours per week

**5. In your staff position, do you typically have direct interaction or contact with patients?**

a. YES, I typically have direct interaction or contact with patients.

b. NO, I typically do NOT have direct interaction or contact with patients.

**ANTICIPATED TURNOVER SCALE**

(Hinshaw, A.S. and Atwood, J.R.)

Response Options

AS = Agree Strongly

MA = Moderately Agree

SA = Slightly Agree

U = Uncertain

SD = Slightly Disagree

MD = Moderately Disagree

DS = Disagree Strongly

| Directions: For each item below, circle the appropriate response. Be sure to use the full range of responses (Agree Strongly to Disagree Strongly)  **Options** | **Item** |
| --- | --- |
| AS MA SA U SD MD DS | 1. I plan to stay in my position awhile. |
| AS MA SA U SD MD DS | 2. I am quite sure I will leave my position in the foreseeable future. |
| AS MA SA U SD MD DS | 3. Deciding to stay or leave my position is not a critical issue for me at this point in time. |
| AS MA SA U SD MD DS | 4. I know whether or not I'll be leaving this agency within a short time. |
| AS MA SA U SD MD DS | 5. If I got another job offer tomorrow, I would give it serious consideration. |
| AS MA SA U SD MD DS | 6. I have no intentions of leaving my present position. |
| AS MA SA U SD MD DS | 7. I've been in my position about as long as I want to. |
| AS MA SA U SD MD DS | 8. I am certain I will be staying here awhile. |
| AS MA SA U SD MD DS | 9. I don't have any specific idea how much longer I will stay. |
| AS MA SA U SD MD DS | 10. I plan to hang on to this job awhile. |
| AS MA SA U SD MD DS | 11. There are big doubts in my mind as to whether or not I will really stay in this agency. |
| AS MA SA U SD MD DS | 12. I plan to leave this position shortly. |

1. Do you intend to leave your position in the next 6 months? Yes__ No__

2. Do you intend to leave the nursing profession in the next 6 months? Yes__ No__
